# Wisdom of the Crowd: insights gained from comparing predicted and observed effects of blood pressure lowering strategies

**DOI:** 10.1038/s41371-023-00816-y

**Published:** 2023-04-11

**Authors:** Sonali R. Gnanenthiran, Vivekanand Jha, Abdul Salam, Emily Atkins, Clara K. Chow, Mark R. Nelson, Mike Rakotz, Markus P. Schlaich, Aletta E. Schutte, Tim Usherwood, Anthony Rodgers

**Affiliations:** 1 The George Institute for Global Health, University of New South Wales, Sydney, NSW Australia; 2 The George Institute for Global Health, University of New South Wales, New Delhi, India; 3 School of Public Health, Imperial College, London, UK; 4 Prasanna School of Public Health, Manipal Academy of Higher Education, Manipal, India; 5 The George Institute for Global Health, University of New South Wales, Hyderabad, India; 6 Westmead Applied Research Centre, Faculty of Medicine and Health, University of Sydney, Westmead Hospital, Westmead, NSW Australia; 7 Menzies Institute for Medical Research, University of Tasmania, Hobart, TAS Australia; 8 American Medical Association, Chicago, IL USA; 9 Dobney Hypertension Centre, Medical School, Royal Perth Hospital Unit, University of Western Australia, Perth, WA Australia

**Keywords:** Hypertension, Risk factors

## Abstract

In a first of its kind assessment in cardiovascular research, we assessed whether pooled cardiovascular expertise could accurately predict efficacy and tolerability for both a novel and an established treatment option. A survey was administered prior to the publication of the QUARTET (A Quadruple UltrA-low-dose tReatment for hypErTension) trial. QUARTET was a multicentre, double-blind, parallel-group, trial that randomised participants to initial treatment with either monotherapy or an ultra-low dose quadruple single pill combination for 12 weeks. Survey participants were asked to predict blood pressure (BP) at 12 weeks and 52 weeks for both groups.

## To the Editor:

Control of high BP is poor globally, with only one third of treated patients achieving BP goals [1, 2]. Under-treatment of high BP with monotherapy is a significant factor underlying this treatment gap [3]. In making complex judgements about expected benefits and risk of treatment, clinicians base decisions on knowledge of trial evidence and personal clinical experience. The ability of clinicians and/or researchers to accurately predict the benefits and adverse effects of BP lowering therapies has not been established, but this may impact upon prescribing of both established and novel BP lowering strategies.

The QUARTET trial randomised participants with hypertension to initial treatment with either monotherapy (irbesartan 150 mg) or to an ultra-low dose quadruple single pill combination (quadpill: irbesartan 37.5 mg, amlodipine 1.25 mg, indapamide 0.625 mg and bisoprolol 2.5 mg) [4]. An online survey (Supplement) was conducted from August 15–29^th^ 2021 prior to the QUARTET trial [4] results being presented at the European Society of Cardiology conference on August 30^th^ 2021. Eligible survey participants (self-identified clinicians or cardiovascular researchers) with experience and/or expertise in high BP voluntarily responded to an email distributed via established mailing lists (Cardiac Society Australia and New Zealand [~500 recipients], Indian Society of Nephrology [~2000 recipients]), and general practice clinician mailing lists [~200 clinicians]), with a link to complete the survey. The survey collected baseline demographic data; prescribing practices (if applicable) and attitudes to BP-lowering drugs combination therapies; estimates of the BP-lowering efficacy and safety (adverse events leading to withdrawal and symptomatic hypotension) of both the quadruple pill and initial monotherapy groups in the QUARTET trial. The survey was administered using Research Electronic Data Capture tools hosted by The George Institute for Global Health, and approved by Sydney Local Health District Ethics Review Committee (2021/ETH010897).

A total of 287 participants responded to the survey. The mean age of the participants was 47 ± 13 years, and 75% were males. The majority were medical practitioners (93%). Of those recruited, 46% were nephrologists, 15% were cardiologists, and 10% were general practitioners. Participants were from across the world, including Asia (46%, predominantly India), Australia/New Zealand (37%), North America (9%), Europe (5%), and Africa (2%). Two-thirds of participants reported no experience with BP clinical trials.

The prediction of systolic BP (SBP) and diastolic BP (DBP) and mean between-group differences by participants was close to that observed in the trial (Table 1). The mean predicted vs observed BP differences were within 1 mmHg of each other at both 12 weeks (predicted: −6/−4 vs observed −5/−3mmHg) and 52 weeks (predicted: −7/−3 vs observed −8/−4mmHg). The observed absolute BP levels were lower than predicted in both the initial monotherapy and quadpill groups at 12 weeks. However, the mean week 52 BP levels were predicted almost exactly - initial monotherapy: predicted 136/80 mmHg vs observed 136/79 mmHg; initial quadpill group: predicted 129/77 mmHg vs observed 128/75 mmHg. There was high agreement that the quadpill was likely to be more effective at lowering BP than monotherapy, with 80 and 68% of those surveyed predicting better SBP and DBP response respectively.

**Table 1:** Predicted and observed effects in the QUARTET trial.

	Predicted			Observed		
	Initial monotherapy group	Initial quadpill group	Mean difference	Initial monotherapy group	Initial quadpill group	Mean difference
Clinic mean SBP/DBP, week 12 (mmHg)	140/84	134/80	−6/−4	134/79	129/76	−5/−3
Clinic mean SBP/DBP, week 52 (mmHg)	136/80	129/77	−7/−3	136/79	128/75	−8/−4
Clinic BP < 140/90 mmHg, week 12 (%)	45%	61%	15%	61%	76%	15%
Clinic BP < 140/90 mmHg, week 52 (%)	55%	70%	15%	62%	82%	20%
Symptomatic hypotension week 0–12 (%)^a^	7%	12%	5%	2%	6%	4%
Withdrawal due to AE week 0–12 (%)	7%	11%	4%	2%	4%	2%

^a^placebo-corrected rates of SBP < 100 (not all symptomatic) and dizziness (irrespective of BP level) were 5%.

BP trial experience was the only predictor of estimated SBP difference at 12 weeks (beta-coefficient −0.2, *p* = 0.009) even post adjustment, with people experienced in clinical trials estimating on average a difference of −9 mmHg, while other respondents estimated −6 mmHg (Supplementary Fig. 1A). Age, sex, country of practice, role, clinical experience in using combination therapy, and place of work were not predictive (Supplementary Fig. 1D, Supplementary Fig. 2A, B, D, E).

Participant predictions of treatment withdrawal due to adverse effects (TWAE) were overestimated three-fold in both the monotherapy and quadpill arms (Table 1, one-sample *t*-test *p* < 0.001 for both). There was broad uncertainty about the relative tolerability of the quadpill: half of the participants predicted that the quadpill would result in a higher risk of TWAE vs. monotherapy, but the other half predicted either no difference or that monotherapy would have more TWAE. Predicted TWAE were consistent across specialties and geographic location (Supplementary Fig. 2C, F). Similarly predicted risk of symptomatic hypotension was also more than two-fold higher than actually observed (Table 1).

There was a positive correlation between predicted SBP difference in favour of Quadpill and predicted side effects for Quadpill, both TWAE (*r* = 0.25, *p* = 0.001) and symptomatic hypotension (*r* = 0.31, *p* < 0.001). Overall, 44% of respondents predicted better efficacy and poorer TWAE, while only 10% predicted better efficacy and better TWAE and only 4% predicted worse efficacy and worse TWAE (Supplementary Table 1).

BP trial experience was a univariable predictor of more accurately estimating TWAE, but was no longer predictive post multivariable adjustment for age, sex, country of practice, role, and place of work (Supplementary Fig. 1B, E). However, BP trial experience (beta-coefficient 0.25, *p* = 0.001) and age (beta-coefficient 0.22, *p* = 0.005) remained significant predictors of estimated symptomatic hypotension difference on multivariable analysis. People who were younger and without BP trial experience were more likely to overestimate risks of hypotension (Supplementary Fig. 1C, F). Only previous BP trial experience was a positive predictor of starting with combination therapy in both newly diagnosed (beta-coefficient 1.466, *p* < 0.001) and already treated hypertensive patients (beta-coefficient 1.024, *p* = 0.047) in multivariable analyses.

In medical practitioners who commenced combination BP therapy in >25% of newly diagnosed hypertensive patients, there was no statistically significant difference in predicted BP difference compared to those less likely to use combination therapy. Such prescribers were less likely to overestimate TWAEs and symptomatic hypotension risks than those who used initial combination therapy less frequently (Supplementary Table 2).

In a first of its kind assessment in cardiovascular research, we demonstrated that pooled expert judgments accurately predicted effects of BP-lowering drug therapies, but there was high heterogeneity and large overestimation of side effects. There was high consensus that the quadpill would be more efficacious than monotherapy, but high discrepancy on whether adverse effects would differ and if so in what direction.

There are several limitations of our study. The survey utilised convenience sampling and thus further research is needed to confirm these results across sexes, specialities, and locations. Primary care doctors were relatively underrepresented in the survey. There are limitations to physician surveys inherent in the need to recruit respondents willing and able to give time to answer pre-defined questions. Sampling may therefore not be representative of real practice. However, the strength of this study is its contemporary assessment of pooled expert judgments which offers a unique opportunity to examine gaps and differences in understanding of perceived safety, efficacy, and utility.

The overestimation of adverse effects of the quadpill might be explained by uncertainty concerning the net effects of a greater number of drugs and higher BP reduction (more adverse effects expected), but lower doses of drugs (fewer side effects expected). However the overestimation of adverse effects also occurred for the usual care group of initial monotherapy with which many would be clinically familiar and for which clinical trial evidence shows little or no increase in TWAE compared to placebo [5, 6]. We hypothesise that this may be due to cognitive biases, in particular negativity bias and availability heuristic: undesirable outcomes tend to have greater salience than desirable ones and we tend to overestimate the likelihood of events with greater “availability” in memory, i.e. outcomes that are easier to conceptualise tend to be perceived as more likely to occur [7]. Overestimation of adverse effects was most notable in younger respondents and those without clinical trial experience, which may be consistent with risk estimates being driven by inexperience. Such perceptions may impact upon clinical care, influencing both prescribing and patients’ receptivity to therapies, as seen in other medical discplines [8]. An inaccurate ability to estimate benefits vs. risks may predispose to underutilisation of combination therapies and low uptitration rates [9], further exacerbating undertreatment of hypertension. Treatment inertia is frequently driven by uncertainty around “usual” BP levels for the practitioner [10, 11], leading to a reluctance to initiate or intensify treatment. Future research should focus more on adverse effects and strategies to address cognitive biases when integrating expected effects, clinical experience, and evidence from randomised trials.

## Summary

### What is known about the topic

- Control of high BP is poor globally, with only one third of treated patients achieving BP goals
- Under-treatment of high BP with monotherapy is a significant factor underlying this treatment gap
- The ability of clinicians and/or researchers to accurately predict the benefits vs. adverse effects of BP lowering therapies has not been established, but may impact upon prescribing of both established and novel BP lowering strategies.

### What this study adds

- There is widespread agreement and a strong ‘wisdom of the crowd’ effect in predicting the efficacy of both novel and established BP lowering regimens.
- Participants’ predictions of adverse effects were highly heterogenous, with adverse effects leading to treatment withdrawal overestimated by 3-fold in both the monotherapy and quadpill arms.
- The high level of uncertainty about tolerability and a tendency to over-estimate side effects may lead to underutilization of BP therapies.

## Supplementary information

Supplement - Information sheet and survey

Supplemental Material
