# Supplementary material for: Wisdom of the Crowd: insights gained from comparing predicted and observed effects of blood pressure lowering strategies
Source: J Hum Hypertens. 2023 Apr 11;37(5):422–4. doi: 10.1038/s41371-023-00816-y (PMC10156589; doi:10.1038/s41371-023-00816-y)
Supplement: Supplementary file 1 — Supplement - Information sheet and survey [file 41371_2023_816_MOESM1_ESM.docx]

**OUTSTANDING ISSUES IN COMBINATION BLOOD PRESSURE LOWERING SURVEY**

**Invitation to Participants**

You are invited to take part in a survey that aims to assess attitudes and practices related to combination blood pressure lowering therapy. You have been contacted as you have relevant expertise and experience in raised blood pressure. In particularly, we would like to see whether experienced clinicians and cardiovascular researchers can predict the outcomes of the QUARTET randomised clinical trial (A Quadruple UltrA-low-dose tReatment for hypErTension, RCT), the results of which will soon be released. The survey is being conducted by the George Institute for Global Health.

The survey will take 5-10 minutes to complete. Your participation in this survey is voluntary and there will be no consequences to you if you do not participate. You can withdraw at any time. While we intend for this survey to further medical knowledge of attitudes and practices related to combination blood pressure lowering therapy, it may not be of direct benefit to you. Completing this survey also has no risks to yourself. All responses are anonymous and the results will be reported in summary form.

The survey results may be published in scientific journals. This survey has been reviewed and approved by the Sydney Local Health District (Royal Prince Alfred) Human Research Ethics Committee (2021/ETH010897). Data may be used for future use, and shared locally and internationally with other research collaborators however ethics approval will be sought prior. The data will be analysed and stored on a research database held within the George Institute for Global Health, 1 King St, Newtown, Sydney, New South Wales. If you consent to participate in this survey, please press on the link below to continue.

‘Begin Survey

**Questionnaire**

***A little about yourself***

1. What is your professional role? Please tick all that apply.

- Medical practitioner
  - General practitioner
  - Cardiologist
  - Nephrologist
  - Endocrinologist
  - General medicine
  - Physician in training e.g. medical registrar
  - Other
- Cardiovascular researcher/scientist
- Other

1. Where do you work?

- Australia
- New Zealand
- Asia
- North America
- South America
- United Kingdom
- Europe
- Africa
- Other

1. What sex do you identify with?

- Male
- Female
- Other

1. How old are you?

---- (insert number) years

1. How do you spend most of your working week?

- Private practice
- Public practice
- Academic institution
- Other (state)

1. Do you have any experience in the design and/or conduct of high blood pressure clinical trials?
   1. No
   2. Yes

***Your predictions for the QUARTET trial results***

Please review the QUARTET (Quadruple UltrA-low-dose tReatment for hypErTension) trial details below before answering the questions.

| **Participants** | Adults with hypertension recruited from primary care practices & hospital outpatient clinics in Australia:   - Untreated with office blood pressure (BP) 140-179/90-99 mmHg or ambulatory daytime BP ≥135/85 mmHg, OR - Monotherapy treated with office BP 130-179/85-99 mmHg or ambulatory daytime SBP ≥125/80 mmHg   At baseline, half were untreated, half were on monotherapy.  All started/switched to one of 2 treatments as outlined below (double blinded). | | |
| --- | --- | --- | --- |
| **Intervention group** | Initial QUADPILL:  Ultra-low dose quadruple single pill combination (irbesartan 37.5 mg, amlodipine 1.25 mg, indapamide 0.625 mg and bisoprolol 2.5 mg) |  | option to add amlodipine 5 mg from week 6 onwards and other therapy from week 12 onwards if office BP>140/90 mmHg |
| **Control group** | Initial MONOTHERAPY:  Irbesartan 150 mg |  |  |
| **Outcomes** | Differences between groups in change in mean BP, BP control and tolerability. | | |
| **Treatment duration** | Primary outcome at 12 weeks, extended follow-up to 52 weeks | | |

Baseline mean office BP was **153/89 mmHg** in both study groups. Considering the trial characteristics noted above, please estimate the efficacy and tolerability of the two treatments approaches in the table below.

|  | Initial Quadpill group | Initial monotherapy group |
| --- | --- | --- |
| Clinic mean SBP/DBP at week 12 (mmHg) | / | / |
| Clinic mean SBP/DBP at week 52 (mmHg) | / | / |
| Participants with clinic BP <140/90 mmHg at week 12 (%) | % | % |
| Participants with clinic BP <140/90 mmHg at week 52 (%) | % | % |
| Participants with symptomatic hypotension between baseline and week 12 (%) | % | % |
| Participant withdrawal due to adverse effects between baseline and week 12 (%) | % | % |

 SBP systolic blood pressure; DBP diastolic blood pressure

1. In terms of reasons that prescribers would not intensify therapy at week 6 week for trial participants with BP above 140/90 mmHg, please estimate how frequently the following reasons would be cited.

|  | **Common** | **Less common** | **Rare** | **Never** |
| --- | --- | --- | --- | --- |
| BP is close to target, with further BP reduction possible given long time needed for drugs to reach full efficacy |  |  |  |  |
| BP regarded as acceptably close to target |  |  |  |  |
| Systolic BP target met, high diastolic BP is less important |  |  |  |  |
| Patient preference |  |  |  |  |
| Not aware of BP target <140/90 |  |  |  |  |
| Concern about potential side effects |  |  |  |  |
| Other, please specify: ----------- [box to enter free text] |  |  |  |  |

1. Of the trial participants with BP above the target BP of 140/90mmHg at week 6, what proportion do you think received intensification of therapy with the addition of amlodipine 5 mg?

- 0-20%
- 21-40%
- 41-60%
- 61-80%
- 81-100%

***Blood Pressure (BP) Prescribing Practices***

1. In your own practice, for what proportion of patients *starting treatment for hypertension* do you start with combination therapy (ie. more than one blood pressure lowering medicine as 1^st^ line)?

- 0%
- 1-25%
- 26-50%
- 51-75%
- >75%
- Not applicable: I do not prescribe medications

1. In what proportion of your patients *already treated for hypertension* do you start treatment with combination therapy (ie. use as 2^nd^, 3^rd^ line etc)?

- 0%
- 1-25%
- 26-50%
- 51-75%
- >75%
- Not applicable: I do not prescribe medications

1. For initial (1^st^ line) pharmacological treatment of hypertension, in what situations do you currently use combination therapy? ***You can choose more than one answer***.

- None
- >20/10 mmHg above target
- High cardiovascular disease risk
- Potential adherence issues
- Other
- Not applicable: I do not prescribe medications

1. For initial (1^st^ line) pharmacological treatment of hypertension, please select your reasons for not using combination therapy. ***You can choose more than one answer***.

- Not supported by randomized clinical trials
- Not recommended by guidelines
- Lack of need (e.g. if not controlled on monotherapy can be uptitrated at next visit)
- Physician inexperience in using combination therapy
- Risk of adverse effects
- Limited availability of suitable combination therapy medications on the market
- Other
  - Please specify ----------- [box to enter free text]

1. What do you see as the key research question(s) related to understanding the role of low-dose combination therapy in clinical care of people with hypertension?

----------- [box to enter free text]

**Thank you for your participation.**
