# Supplementary material for: Wisdom of the Crowd: insights gained from comparing predicted and observed effects of blood pressure lowering strategies
Source: J Hum Hypertens. 2023 Apr 11;37(5):422–4. doi: 10.1038/s41371-023-00816-y (PMC10156589; doi:10.1038/s41371-023-00816-y)
Supplement: Supplementary file 2 — Supplemental Material [file 41371_2023_816_MOESM2_ESM.docx]

**SUPPLEMENTAL FILE**

|  | **Better efficacy** | **Same efficacy** | **Worse efficacy** | **P**  **value** |
| --- | --- | --- | --- | --- |
| **Better AE with quadpill – no. (%)** | 17 (10) | 4 (2) | 7 (4) | 0.003 |
| **Same AE with quadpill – no. (%)** | 42 (25) | 9 (5) | 3 (2) |  |
| **Worse AE with quadpill – no. (%)** | 74 (44) | 8 (5) | 3 (2) |  |

**Supplemental Table 1: Proportion of Survey Responders according to predictions of efficacy for SBP diff and withdrawal due to adverse events (TWAE) at 12 weeks (n=167)**

AE: adverse effects

**Supplemental Table 2: Predicted Adverse Effect Profile leading with Trial Withdrawal According to Use of Combination Blood Pressure Lowering Therapy (n=156).**

|  | **Use of Combination Blood Pressure Therapy when starting BP therapies** | | **P-Value** |
| --- | --- | --- | --- |
|  | **0-25%** | **>25%** |  |
| **Better AE with quadpill** – no (%) | 17 (17) | 8 (14) | 0.01 |
| **Same AE with quadpill** – no (%) | 22 (22) | 25 (45) |  |
| **Worse AE with quadpill**– no (%) | 61 (61) | 23 (41) |  |

AE: adverse effect

**Supplemental Figure 1: Quad pill vs monotherapy - predicted vs observed differences in blood pressures and adverse events stratified by BP trial experience and age.**

(A) Predicted difference in SBP stratified by BP trial experience (mmHg, standard deviation [SD]); (B) Predicted difference in adverse events leading to withdrawal stratified by BP trial experience (%, SD); (C) Predicted difference in symptomatic hypotension stratified by BP trial experience (%, SD); (D) Predicted SBP across age groups (mmHg, SD); (E) Predicted difference in adverse events leading to withdrawal across age groups (%, SD); (F) Predicted adverse events across age groups (%, SD).

**
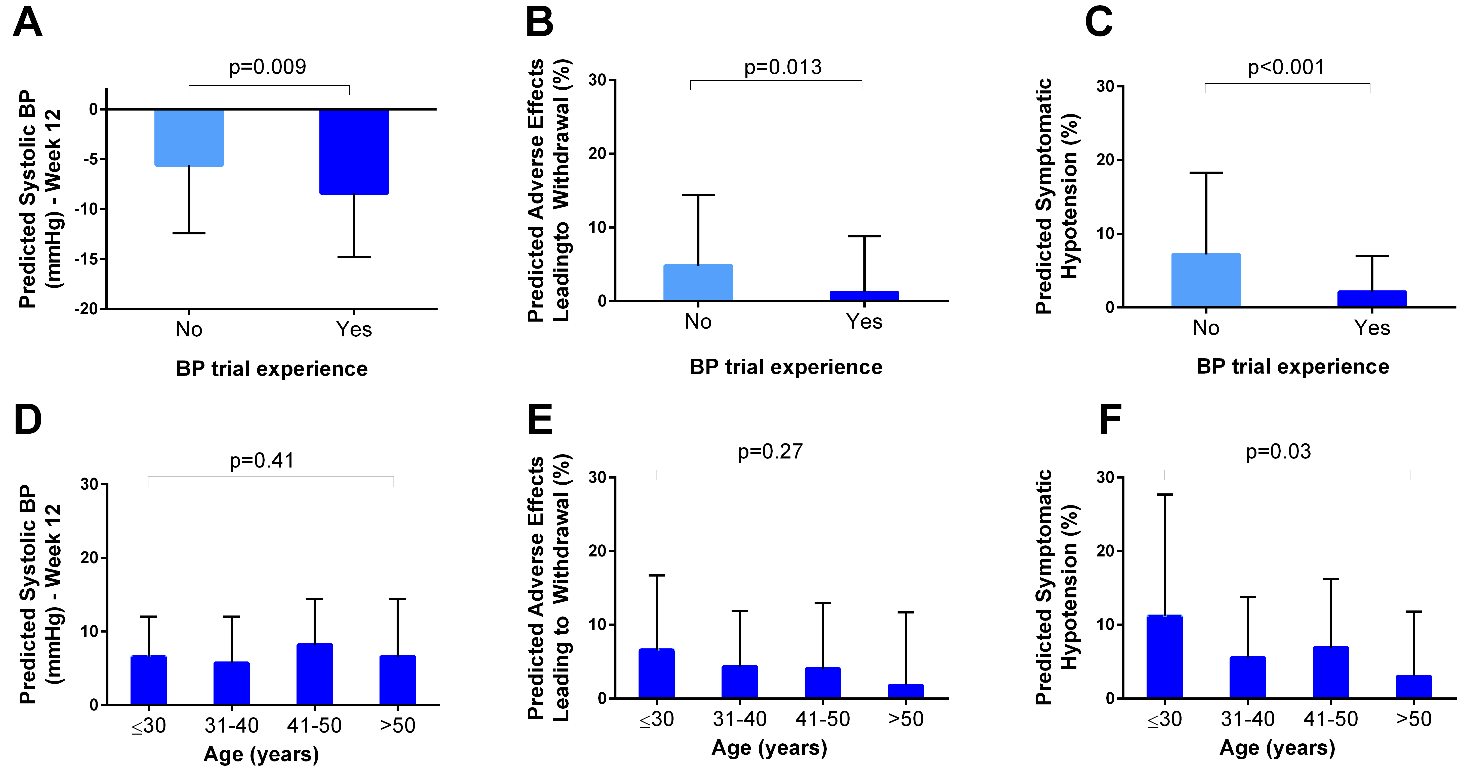
**

**Supplemental Figure 2: Predicted vs observed differences in blood pressures and adverse events leading to withdrawal**

(D) Predicted SBP across specialties (mmHg); (E); Predicted DBP across specialties (mmHg); (F) Predicted adverse events across specialties (%); (G) Predicted SBP across geographic locations (mmHg); (H) Predicted DBP across geographic locations (mmHg); (I) Predicted adverse effects across geographic locations (%).

**
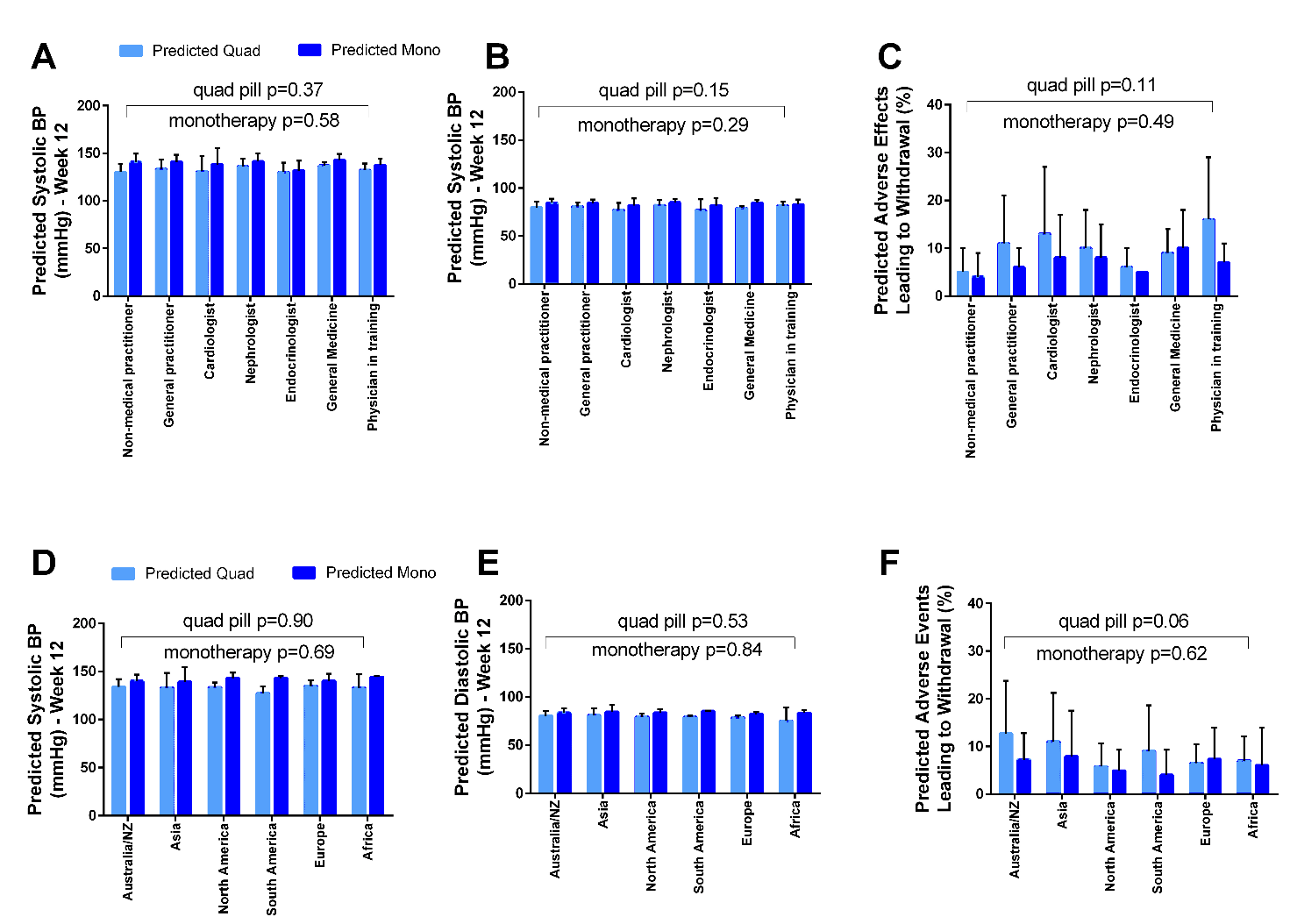
**
